# Huge pseudoaneurysm of the left ventricular outflow tract developed a few years after surgery for subvalvular mitral aneurysm: to plug or not to plug it is the question

**DOI:** 10.1093/ehjimp/qyae002

**Published:** 2024-01-18

**Authors:** Humberto Morais, Elsa Fernandes, Mauer A A Gonçalves

**Affiliations:** Centro de Estudos Avançados em Educação e Formação Médica, Faculdade de Medicina, da Universidade Agostinho Neto, Avenida Hoji-Ya-Henda, Luanda, Angola; Cardiology Department, Hospital Militar Principal/Instituto Superior, Rua Pedro Miranda 40-42 Maianga, Luanda, Angola; Cardiology Service, Clínica Girassol, Luanda, Angola; Centro de Estudos Avançados em Educação e Formação Médica, Faculdade de Medicina, da Universidade Agostinho Neto, Avenida Hoji-Ya-Henda, Luanda, Angola; Cardiology Service, Clinical Medical Center, Luanda, Angola

**Figure:**
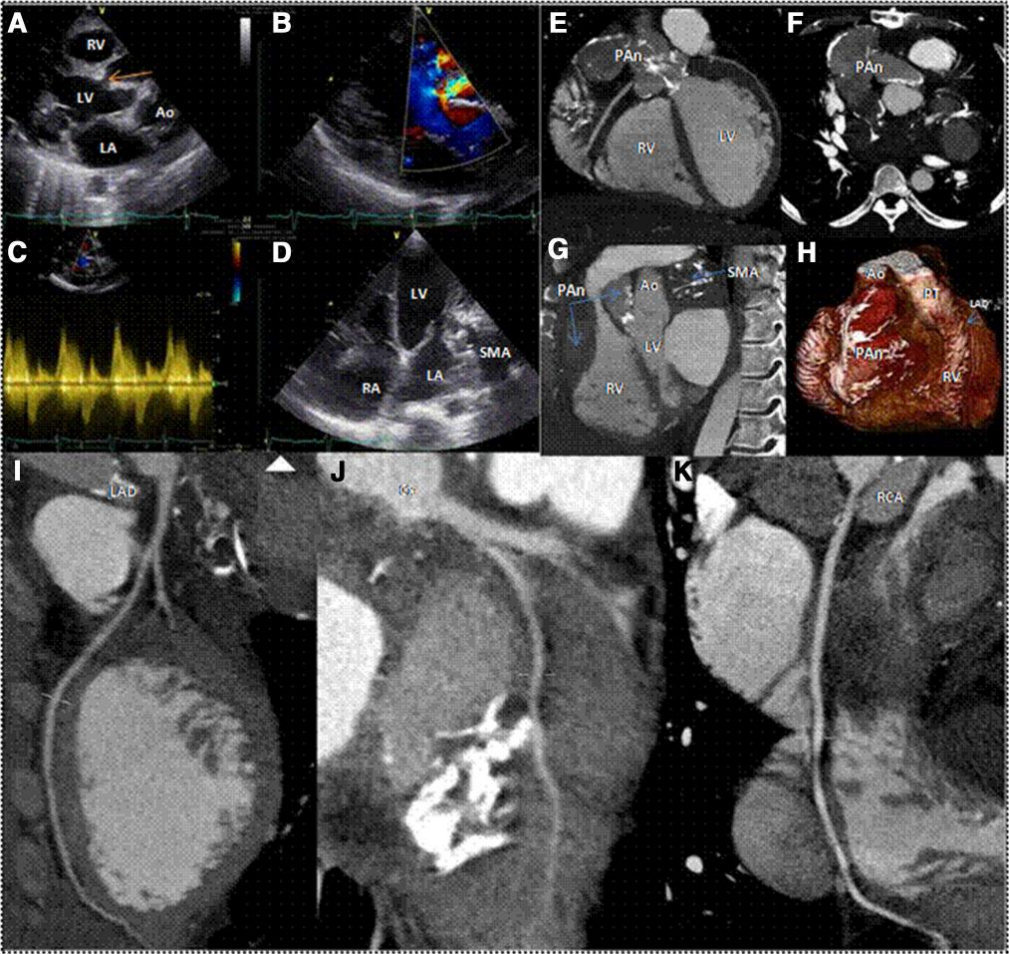


A 36-year-old male patient presented with dyspnoea, fatigue, and pre-syncope episodes during moderate exertion. Relevant history included surgery for a subvalvular mitral aneurysm (SMA) performed 14 years earlier. Although surgery was done suspecting a type A aortic aneurysm, the diagnosis was not confirmed intraoperatively. Detailed surgical notes were not available. Physical examination revealed a systolic-bar murmur in the third left intercostal space. 2D-transthoracic echocardiography and Doppler study displayed a large structure linked to the left ventricle through a narrow neck (*Panel A*, arrow), showing a turbulent jet filling a pseudoaneurysm (*Panel B*) with a high gradient between the ventricle and the pseudoaneurysm (*Panel C*). An operated SMA aneurysm was also observed (*Panel D*).

A 64-slice gated CT (*Panels E*, *F*, and *G*, Supplementary data online, *Movies S1*, *S2*, and *S3*) with volume rendering (*Panel H*) confirmed the presence of SMA (*Panel G*) and also showed a massive pseudoaneurysm from the left ventricular outflow tract (LVOT), impacting the right atrioventricular groove and the ascending aorta, compressing the right ventricle (*Panels G* and *H*).

Both the LVOT pseudoaneurysm and SMA shape the atrioventricular groove without evident coronary artery compression (*Panels I*, *J*, and *K*). This image spotlight exemplifies the case of a patient with a long-standing LVOT pseudoaneurysm. LVOT pseudoaneurysms are rare, often post-infective endocarditis or cardiac surgery. Surgical repair is generally advised, but percutaneous closure (PC), when feasible, may be preferred in high-risk cases. However, due to PC unavailability and high surgical risk, the patient was managed conservatively with furosemide 40 mg twice a day; losartan 25 mg once a day, spironolactone 25 mg once a day, carvedilol 6.25 mg twice a day. He is currently in NYHA Class II.

Ao, aorta; Cx, circumflex artery; LA, left atrium; LAD, left anterior descending artery; LV, left ventricle; PAn, pseudoaneurysm; RCA, right coronary artery; RA, right atrium; RV, right ventricle.

## Supplementary Material

qyae002_Supplementary_Data

## Data Availability

The article’s data will be shared on reasonable request to the corresponding author.

## Supplementary data

**Consent:** Informed consent was given by the patient.

**Funding:** None declared.

## Lead author biography

My name is Humberto Morais, I am a cardiologist. I graduated from the Leningrad Military Medical Academy, in the Union of Soviet Socialist Republics, in 1986. For many years, I was head of the Cardiology Department at the Main Military Hospital/Higher Institute of Angola. Currently, I am a guest researcher at the Center for Advanced Studies in Medical Education (CEDUMED) at the Faculty of Medicine of the Dr. António Agostinho Neto University, Luanda, Angola, and a medical consultant at Clínica Girassol, Luanda, Angola.

